# Bentall procedure in Takayasu arteritis

**DOI:** 10.1186/1749-8090-8-S1-P17

**Published:** 2013-09-11

**Authors:** M Hidiroglu, A Kucuker, L Cetin, H Bayram, F Sağlam, E Sener

**Affiliations:** 1 Cardiovascular Surgery Department, Ataturk Training and Research Hospital, Ankara, Turkey

## Background

Takayasu arteritis is an idiopathic chronic inflammatory disease affecting frequently the aorta-big arteries and may cause stenosis, occlusion or aneursymatic degeneration. Coarctation or aneurysm formation may be seen due to aortitis. We present a patient with severe aortic regurgitation and ascending aorta dilatation we treated surgically.

## Methods

A 38 years old man with diagnose of Takayasu arteritis was admitted to our hospital with chest pain. Echocardiography revealed severe aortic regurgitation + ascending aorta dilatation. Computed tomographic angiography showed ascending aorta was 44 mm. He was scheduled for elective cardiac operation. His medication was adjusted as Addison protocol for operation. Axillary artery cannulation and median sternotomy was performed. Ascending aorta was aneursymatic up to truncus brachiocephalicus. Coronary ostia were prepared as buttons. Aortic valve and ascending aorta was resected and replaced with a valved conduit including a 25 # St Jude mechanical prosthetic valve and 30 mm Dacron tube graft. The left and the right coronary ostia were anastomosed on the Dacron tube graft. Bentall procedure was completed with the distal anastomosis of Dacron graft strengthened with Teflon strip to aorta beyond truncus cephalicus. Cardiopulmonary bypass was terminated without inotropic support.

## Results

Intensive care unit and hospital follow-up was uneventful. He was discharged with low dose corticosteroids at postoperative sixth day. Although surgical therapies for patients with chronic vasculitis may be challenging due to chronic inflammatory influences on vessels, a meticulous approach with supporting the anastomosis with additive surgical materials may improve surgical success.

## Conclusion

Although surgery in Takayasu arteritis is known to be complicated because of vessel structure due to chronic inflammation. A radical and elaborate approach may ease an uneventful operation when surgery is mandatory.

